# Controlling neuropathic pain by adeno-associated virus driven production of the anti-inflammatory cytokine, interleukin-10

**DOI:** 10.1186/1744-8069-1-9

**Published:** 2005-02-25

**Authors:** Erin D Milligan, Evan M Sloane, Stephen J Langer, Pedro E Cruz, Marucia Chacur, Leah Spataro, Julie Wieseler-Frank, Sayamwong E Hammack, Steven F Maier, Terence R Flotte, John R Forsayeth, Leslie A Leinwand, Raymond Chavez, Linda R Watkins

**Affiliations:** 1 Department of Psychology & the Center for Neuroscience, University of CO at Boulder, Boulder, CO 80309 USA; 2 Department of Molecular, Cellular & Developmental Biology, University of CO at Boulder, Boulder, CO 80309 USA; 3 Genetics Institute, the Powell Gene Therapy Center & Department of Pediatrics, University of FL at Gainesville, Gainesville, FL 32610 USA; 4 Avigen, Inc., Alameda, CA 94502 USA

## Abstract

Despite many decades of drug development, effective therapies for neuropathic pain remain elusive. The recent recognition of spinal cord glia and glial pro-inflammatory cytokines as important contributors to neuropathic pain suggests an alternative therapeutic strategy; that is, targeting glial activation or its downstream consequences. While several glial-selective drugs have been successful in controlling neuropathic pain in animal models, none are optimal for human use. Thus the aim of the present studies was to explore a novel approach for controlling neuropathic pain. Here, an adeno-associated viral (serotype II; AAV2) vector was created that encodes the anti-inflammatory cytokine, interleukin-10 (IL-10). This anti-inflammatory cytokine is known to suppress the production of pro-inflammatory cytokines. Upon intrathecal administration, this novel AAV2-IL-10 vector was successful in transiently preventing and reversing neuropathic pain. Intrathecal administration of an AAV2 vector encoding beta-galactosidase revealed that AAV2 preferentially infects meningeal cells surrounding the CSF space. Taken together, these data provide initial support that intrathecal gene therapy to drive the production of IL-10 may prove to be an efficacious treatment for neuropathic pain.

## Background

Neuropathic pain is an especially difficult chronic pain syndrome to treat. No compounds are yet available that successfully resolve such pain [1-3]. To date, drug therapies developed for human neuropathic pain have targeted neurons. However, evidence has recently accumulated that pathological pain, including neuropathic pain, is dynamically and dramatically amplified as a result of spinal cord glial activation [4-6]. Spinal cord glia become activated as a consequence of inflammation and/or trauma to peripheral nerves [4,6]. This raises the intriguing possibility that finding ways to target glial activation, or its downstream consequences, may provide a novel approach for neuropathic pain control [5,7].

Several glial-selective drugs have been successful in preventing or reversing neuropathic pain in animals, but none is optimal for clinical applications. For example, fluorocitrate is a selective astrocyte inhibitor [8,9]. While effective in blocking the induction of neuropathic pain in animals [10], fluorocitrate is inappropriate for human use due to inhibition of glial glutamate uptake and consequent seizures can occur [11]. Similarly, minocycline is a selective microglial inhibitor [12]. It too is effective in blocking the induction of neuropathic pain in animals [13,14]. However, as minocycline fails to affect established neuropathic pain [13,14], this compound does not appear to have therapeutic potential.

Other approaches have focused on the fact that glia are "immune cell-like". Upon activation, glia and immune cells each release pro-inflammatory substances, most notably pro-inflammatory cytokines (interleukin [IL]-1, tumor necrosis factor [TNF] and IL-6). The release of pro-inflammatory cytokines by activated spinal cord glia is key as these cytokines enhance pain and have been implicated in the initiation and maintenance of neuropathic pain [10,15,16]. Immunosuppressive (methotrexate) and immunomodulatory (propentofylline) drugs have been tested with the aim of suppressing neuropathic pain via suppression of glial-derived pro-inflammatory cytokines in spinal cord. While these drugs have proven effective in reducing enhanced pain responses [17,18], they are not optimal for chronic use in humans, as their systemic administration would negatively impact the peripheral immune system. In addition, although selective pro-inflammatory cytokine antagonists have proven successful in resolving neuropathic pain [10,15], their lack of CNS penetration negates systemic administration and their relatively short duration of action poses problems for chronic intrathecal administration in humans.

The purpose of the present series of studies was to explore a new approach to neuropathic pain control; that is, intrathecal gene therapy using an adeno-associated viral (serotype II; AAV2) vector to drive the production and release of interleukin-10 (IL-10), a powerful *anti*-inflammatory cytokine [19]. As IL-10 is known to suppress the production and release of all 3 pro-inflammatory cytokines [19], it would be predicted to be efficacious for the treatment of neuropathic pain. Two animal models of neuropathic pain were employed: (a) sciatic inflammatory neuropathy (SIN) induced by localized inflammation of the sciatic nerve in the absence of frank trauma, and (b) chronic constriction injury (CCI), a model involving both trauma to and inflammation of the sciatic nerve. Depending upon the intensity of sciatic nerve inflammation induced, the SIN model can produce either an ipsilateral or bilateral mechanical allodynia [20]. This allows examination of both ipsilateral and mirror-image pain. CCI was chosen for study both because it is a classic model of partial nerve injury [21] and because it induces thermal hyperalgesia in addition to mechanical allodynia [22-24]. We attempted to prevent and reverse SIN- and CCI-induced pain changes, respectively. These studies demonstrate that intrathecal administration of an AAV2 vector that encodes for rat IL-10 (AAV2-r-IL-10) is effective in transiently resolving neuropathic pain in both models.

## Results

### AAV transgene expression *in vitro* and *in vivo*

The plasmid construct containing rat IL-10 cDNA (pTR2-CB-rIL-10) was transfected into an IB3 cell line to verify plasmid-induced rat IL-10 release (see Methods). Media from the transfected cell cultures were collected 18, 36, and 60 hr later and frozen at -80°C until analyzed for rat IL-10 by ELISA. Rat IL-10 was readily detected in culture supernatants of transfected cells versus untransfected control cultures (see Fig. 2A Inset). This construct was subsequently used to create an AAV2 vector for *in vivo *testing.

**Figure 1:**
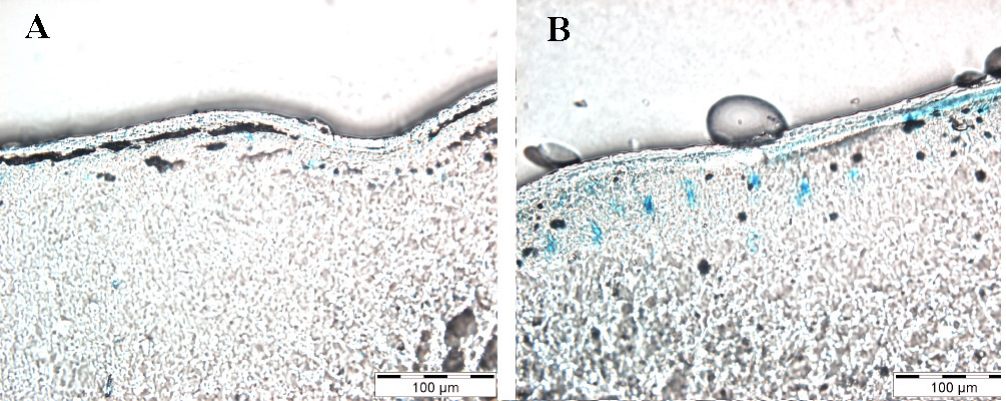
**Photomicrographs of beta-Galactosidase histochemistry; expression in spinal cord meninges. **Spinal cords were removed from control, non-injected rat (A) or the AAV2-LacZ injected rat (B) 8 days after intrathecal injection. Beta-Galactosidase histochemistry was conducted with X-gal staining procedures. No sections are counter stained. Magnification in Panels A and B are identical, scale bar, 100 microns.

**Figure 2:**
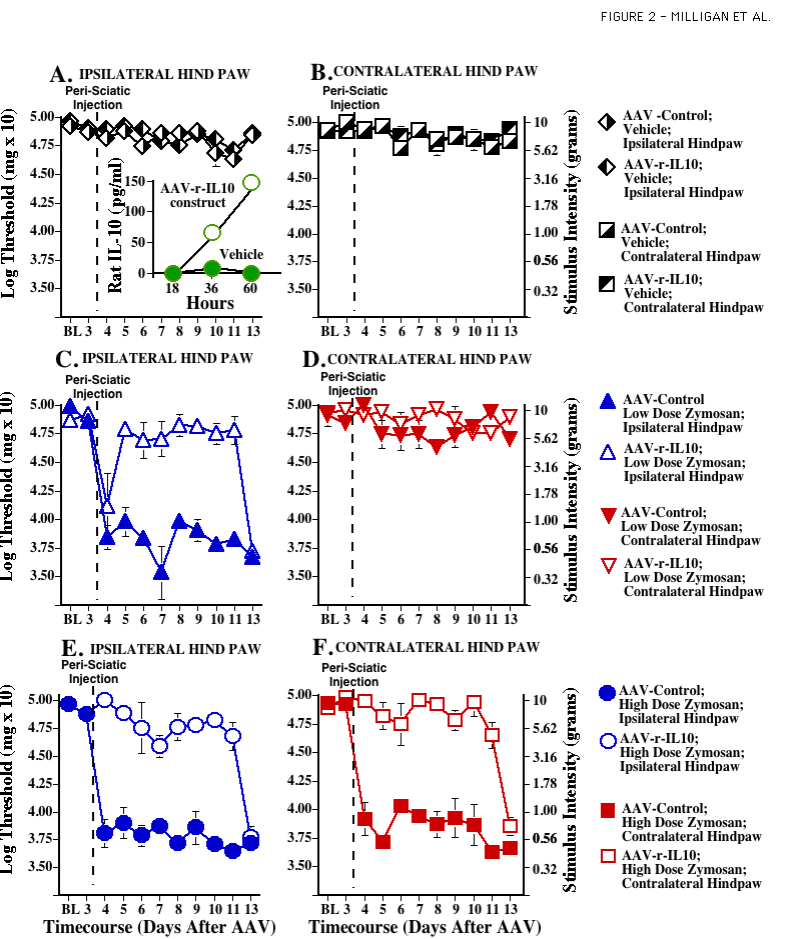
**Adeno-associated viral IL-10 blocks development of chronic sciatic inflammatory neuropathy (SIN) induced mechanical allodynia. **After baseline (BL) assessment on the von Frey test, all rats received intrathecal AAV2-GFP (Control, encoding green fluorescent protein) or AAV2-r-IL-10. Behavior was reassessed Day 3 after intrathecal AAV, confirming that neither AAV2-GFP (Control) nor AAV2-r-IL-10 affected behavior prior to peri-sciatic injections (F _7,88 _= 0.686, p > 0.68). After this Day 3 assessment, unilateral peri-sciatic injections of 0 (vehicle control; **Panels A, B**), 4 ug zymosan (to induce ipsilateral allodynia; **Panels C, D**), or 160 ug zymosan (to induce bilateral allodynia; **Panels E, F**) were delivered, with repeated re-administration across days to induce a chronic neuropathic state. Repeated measures ANOVA revealed reliable main effects of peri-sciatic zymosan dose (F _1,40 _= 12.093, p < 0.002), IL-10 (F _1,40 _= 69.829, p < 0.0001), and laterality (F _1,40 _= 22.315, p < 0.0001), and interactions between zymosan dose and IL-10 (F _1,40 _= 6.161, p < 0.02) and between IL-10 and laterality (F _1,40 _= 15.412, p < 0.001). The construct pTR2-CB-r-IL-10 employed in an AAV vector for behavioral testing induced the production and release of rat IL-10 from transfected IB3 cells in culture. Increases in rat IL-10 protein were detected in supernatants of transfected cells versus untransfected vehicle control cultures (**Panel A Inset**). Neither AAV2-GFP nor AAV2-r-IL-10 affected the behavioral responses of rats receiving chronic peri-sciatic vehicle, as illustrated by data obtained from the hindpaws ipsilateral (**Panel A**) or contralateral (**Panel B**) to the peri-sciatic injections. Allodynia was induced in the ipsilateral hindpaw of intrathecal AAV2-GFP rats receiving 4 ug peri-sciatic zymosan (**Panel C**). This allodynia was largely blocked by AAV2-r-IL-10 (p > 0.045 through Day 11 compared to BL) with allodynia reappearing on Day 13 after AAV; that is, 10 days after initiation of chronic zymosan. Again, neither AAV2-GFP nor AAV2-r-IL-10 affected behaviors obtained from the contralateral, non-allodynic hindpaws (**Panel D**). Allodynia was induced in both the ipsilateral (**Panel E**) and contralateral (**Panel F**) hindpaws of intrathecal AAV2-GFP rats receiving 160 ug peri-sciatic zymosan. These ipsilateral and contralateral allodynias were largely blocked by AAV2-r-IL-10 (p > 0.15 through Day 11 compared to BL), until allodynia reappeared on Day 13 after AAV; that is, 10 days after initiation of chronic zymosan.

Lumbosacral intrathecal injection of adenovirus has been well characterized as infecting predominantly meningeal cells surrounding the lower spinal cord CSF space [25]. A comparable examination of AAV2 spread has not been previously reported in adult rats following lumbosacral intrathecal injection. Rats (n = 3) were injected intrathecally with AAV2 (approximately 4.1 × 10^8 infectious particles in 10 ul) engineered to express beta-galactosidase 8 days prior to tissue fixation for beta-galactosidase histochemistry. Tissues from non-injected control rats (n = 2) were processed at the same time.

A diffuse and sporadic pattern of beta-galactosidase expression was observed from the injection site rostral to spinal cord C2 regions with the most robust staining observed closest to the injection site. Close inspection of spinal cord tissue (Fig. 1A) revealed little to no beta-galactosidase expression in control naive tissue. In contrast, clear beta-galactosidase expression was observed in *Lac*Z injected rats (Fig. 1B), as indicated by stained cells in the pial meningeal layer. Beta-galactosidase expression decreased at more rostral regions (not shown). No beta-galactosidase expression was observed in the meninges of ventral spinal cord. The tissue distribution of AAV2-*Lac*Z induced beta-galactosidase expression was observed predominantly in the pial membrane.

### Blockade of SIN-induced mechanical allodynia by intrathecal AAV2-r-IL-10

Sciatic inflammatory neuropathy (SIN) is induced by peri-sciatic injection of an immune activator, such as zymosan (yeast cell walls), around one healthy sciatic nerve at mid-thigh level [10,20]. This procedure creates robust mechanical allodynia, but no thermal hyperalgesia [20]. Mechanical allodynia is restricted to the injected limb when low doses of zymosan are used. High doses of zymosan induce allodynia in both the injected hindleg as well as the contralateral (mirror-image) hindleg [10,20]. The zymosan injection is done in unanesthetized, unrestrained rats via a pre-implanted peri-sciatic catheter [26]. This allows for repeated injections of the immune activator across days so to induce chronic neuropathic pain [10]. Prior to induction of sciatic inflammatory neuropathy (SIN), rats were assessed for their responses to low-threshold mechanical stimuli (0.407 – 15.136 gm) applied to the plantar surface of their hindpaws (von Frey test). This was done prior to (baseline; BL) and again on Day 3 after intrathecal AAV2-r-IL-10 (8.5 × 10^8 infectious units in 5 ul; dose based on pilot studies) or AAV2-GFP (Control; 8.45 × 10^8 infectious units in 5 ul; directed the intracellular production of jellyfish green fluorescent protein; GFP). AAV2-r-IL-10 and AAV2-GFP had no effect on mechanical response thresholds measured 3 days after virus delivery, compared to BL (F _7,88 _= 0.686, p > 0.68) (Fig. 2). Hence neither the presence of rat IL-10 nor adeno-associated virus had measurable effects on basal low threshold mechanical responses.

Immediately upon completion of the Day 3 test, all rats were peri-sciatically injected with either 4 or 160 ug zymosan (n = 5–6/group) to induce ipsilateral or bilateral mechanical allodynia, respectively. Zymosan was re-administered to maintain allodynia across the testing period, as previously described [10,20,27]. Behavioral responses on the von Frey test were reassessed daily through Day 11 and lastly on Day13 in accordance with prior studies [10].

AAV2-r-IL-10 and AAV2-GFP had no effect on mechanical response thresholds measured 3 days after virus delivery, compared to BL (Fig. 2). Hence neither the presence of rat IL-10 nor adeno-associated virus had measurable effects on basal pain responses. As in our previous studies [10], low dose zymosan induced a unilateral allodynia (Fig. 2A,B) while higher dose zymosan induced a bilateral allodynia (Fig. 2C,D), compared to BL measures. Both ipsilateral (Fig. 2A) and bilateral (Fig. 2C,D) allodynias were blocked by AAV2-r-IL-10 through Day 11, as von Frey responses after peri-sciatic zymosan did not differ from BL. By Day 13, both ipsilateral and bilateral allodynia returned.

### Reversal of chronic constriction injury (CCI) induced mechanical allodynia and thermal hyperalgesia by intrathecal AAV2-r-IL-10

**Figure 3:**
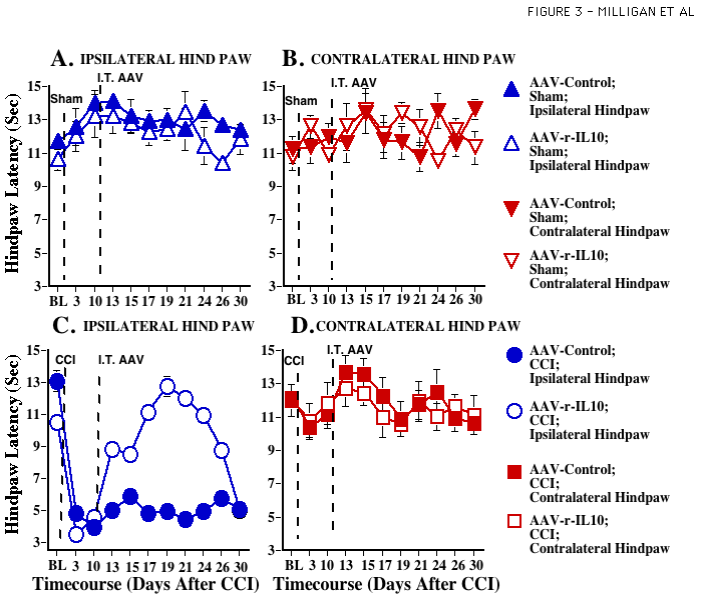
**Adeno-associated viral IL-10 reverses established CCI-induced thermal hyperalgesia. **After predrug (baseline; BL) assessment on the Hargreaves test, sham (**Panels A, B**) or CCI (**Panels C, D**) surgery was performed (timing denoted by the first vertical dotted line). Behavioral assessments were reassessed Days 3 and 10 after surgery to document the lack of thermal hyperalgesia in sham operated rats and development of unilateral allodynia in CCI groups ipsilateral to sciatic surgery. ANOVA revealed reliable main effects of CCI (F _1,40 _= 140.740, p < 0.0001) and laterality (F _1,38 _= 48.901, p < 0.0001), and an interaction between CCI and laterality (F _1,40 _= 104.295, p < 0.0001). After the Day 10 assessment, rats received intrathecal injections of either AAV2-GFP (Control) or AAV2-r-IL-10 (timing denoted by the second vertical dotted line). Behavioral assessments were again recorded on Days 13, 15, 17, 19, 21, 24, 26, and 30 after surgery; that is, Days 3, 5, 7, 9, 11, 14, 16, and 20 days after AAV. While neither AAV2-GFP nor AAV2-r-IL-10 exerted marked effects in sham operated animals (**Panels A, B**) or non-allodynic hindpaws of CCI-operated animals (**Panel D**), AAV2-r-IL-10 transiently reversed ipsilateral CCI allodynia compared to CCI operated AAV2-GFP treated animals (**Panel C**). For Days 13–26, ANOVA revealed reliable main effects of CCI (F _1,39 _= 134.036, p < 0.0001), IL-10 (F _1,39 _= 12.047, p < 0.01) and laterality (F _1,39 _= 66.284, p < 0.0001), and interactions between CCI and AAV2-r-IL-10 (F _1,39 _= 24.486, p < 0.0001), CCI and laterality (F _1,39 _= 91.956, p < 0.0001), and IL-10 and laterality (F _1,39 _= 17.392, p < 0.0001). At Day 30, behavioral responses were not significantly different from Day 10 preinjection levels (F _1,39 _= 7.824, p > 0.10).

**Figure 4:**
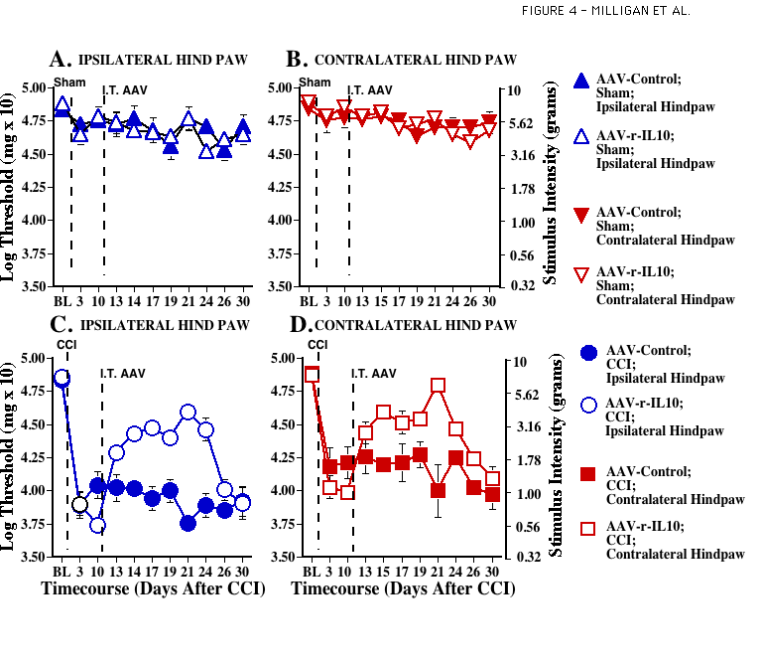
**Adeno-associated viral IL-10 attenuates established CCI-induced mechanical allodynia. **After predrug (baseline; BL) assessment on the von Frey test, sham (**Panels A, B**) or CCI (**Panels C, D**) surgery was performed (timing denoted by the first vertical dotted line). Behavioral assessments were reassessed Days 3 and 10 after surgery to document the lack of allodynia in sham operated rats and development of bilateral allodynia in CCI groups. ANOVA revealed reliable main effects of CCI (F _1,40 _= 197.446, p < 0.0001) and laterality (F _1,40 _= 6.356, p < 0.05). After the Day 10 assessment, rats received intrathecal (i.t.) injections of either AAV2-GFP (Control) or AAV2-r-IL-10 (timing denoted by the second vertical dotted line). Behavioral assessments were again recorded on Days 13, 15, 17, 19, 21, 24, 26, and 30 after surgery; that is, Days 3, 5, 7, 9, 11, 14, 16, and 20 days after AAV. While neither AAV2-GFP nor AAV2-r-IL-10 exerted marked effects in sham operated animals (**Panels A, B**), AAV2-r-IL-10 transiently attenuated bilateral CCI allodynia compared to CCI operated AAV2-GFP treated animals (**Panels C, D**). For Days 13–26, ANOVA revealed reliable main effects of CCI (F _1,40 _= 496.336, p < 0.0001), IL-10 (F _1,40 _= 59.636, p < 0.0001), and laterality (F _1,40 _= 28.565, p < 0.0001), and interactions between CCI and IL-10 (F _1,40 _= 72.988, p < 0.0001) and CCI and laterality (F _1,40 _= 9.325, p < 0.01). At Day 30, behavioral responses were not significantly different from Day 10 preinjection levels (F _1,40 _= 0.696, p > 0.40).

Chronic constriction injury (CCI) is a classic model of neuropathic pain induced by partial nerve injury [21]. Like the SIN model described above, CCI is performed on one sciatic nerve at mid-thigh level. In contrast to the SIN model described above, CCI involves both inflammation of, and trauma to, this nerve, To test whether AAV2-r-IL-10 could reverse established thermal hyperalgesia or low threshold mechanical allodynia induced by CCI, the plantar surface of the rat hindpaws were first assessed for their responses to low threshold mechanical stimuli (von Frey test) and radiant heat stimuli (Hargreaves test) prior to (BL) and again on Days 3 and 10 after surgery. CCI surgery produced reliable ipsilateral thermal hyperalgesia (Fig. 3) and bilateral mechanical allodynia (Fig. 4) compared to sham surgery, in agreement with our prior reports [22-24].

Immediately after behavioral testing on Day 10, rats (n = 5–6 /group) received intrathecal AAV2-r-IL-10 (8.5 × 10^8 infectious units in 5 ul) or AAV2-GFP (8.45 × 10^8 infectious units in 5 ul). Hargreaves and von Frey tests were again performed on Days 3, 5, 7, 9, 11, 14, 16 and 20 after viral administration. This corresponds to Days 13, 15, 17, 19, 21, 24, 26, and 30 after CCI or Sham surgery. The data demonstrate that AAV2-r-IL-10 administration produced significant reversal of both ipsilateral thermal hyperalgesia (Fig. 3) and bilateral allodynia (Fig. 4) induced by CCI, compared to AAV2-GFP, on Days 13–26 after surgery.

Intrathecal AAV2-r-IL-10 did not permanently reverse these ongoing pathological pain states. AAV2-r-IL-10 reversal of CCI-induced pathological pain states began dissipating by Day 26 post-surgery. From Day 26–30, both thermal hyperalgesia and mechanical allodynia progressively returned, with pain facilitation reaching pre-AAV levels by Day 30 (Figs. 3,4). At Day 30, neither behavioral response modality was significantly different from Day 10 preinjection levels.

Lastly, 2 groups of rats (n = 10/group) were matched for body weight prior to receiving intrathecal AAV2-r-IL-10 (8.5 × 10^8 infectious units in 5 ul) or AAV2-GFAP (8.45 × 10^8 infectious units in 5 ul). As part of an unrelated study, these rats were weighed Day 7 post-AAV2 just prior to sacrifice. No difference in the body weight of these 2 groups of animals was found (F _1,18 _= 0.181; p > -.67). As in all prior experiments, no adverse effects of either AAV2-r-IL-10 or AAV-GFAP were noted. All rats appeared healthy, appeared to gain weight normally, and exhibited typical posture, grooming, and locomotion.

## Discussion

The present experiments document the efficacy of AAV2-r-IL-10 in preventing and reversing neuropathic pain. In addition, this work provides initial evidence that intrathecal gene therapy to express anti-inflammatory cytokines, such as IL-10, may be an approach worth pursuing for the treatment of chronic pain. When AAV2-r-IL-10 was administered intrathecally 3 days prior to chronic inflammation of the sciatic nerve (SIN), AAV2-r-IL-10 prevented the onset of both ipsilateral and mirror-image mechanical allodynia, as measured by the von Frey test. Blockade of these SIN-induced allodynias lasted for 8 days (11 days after intrathecal AAV2-r-IL-10), with allodynia developing by day 10 (13 days after intrathecal AAV2-r-IL-10). In contrast, rats receiving AAV2-GFP (Control) prior to induction of SIN exhibited strong ipsilateral and mirror-image allodynia throughout the timecourse tested. Such profound and prolonged allodynia with this chronic SIN procedure is in agreement with prior studies [10]. Neither AAV2-r-IL-10 nor AAV2-GFP altered mechanical response thresholds in sham-operated rats. AA2-r-IL-10 was also successful in reversing established CCI-induced thermal hyperalgesia. Intrathecal AVV2-r-IL-10 administered 10 days after CCI surgery returned hindpaw response latencies to radiant heat (Hargreaves test) to pre-surgery baseline values. The effect of AAV2-r-IL-10 was again transient, with complete reversal observed for a week, from 7 through 14 days after AAV2-r-IL-10. Thermal hyperalgesia then progressively returned with robust hyperalgesia recorded 20 days after AAV-r-IL-10. In contrast, rats receiving intrathecal AAV2-GFP exhibited stable ipsilateral thermal hyperalgesia throughout the timecourse tested. Neither AA2-r-IL-10 nor AAV-GFP altered thermal response thresholds in sham-operated rats. AAV-2-r-IL-10 attenuated established ipsilateral and mirror-image mechanical allodynia in these same animals, with allodynia again becoming fully re-expressed by 16–20 days after AAV2-r-IL-10. Rats receiving intrathecal AAV2-GFP exhibited marked bilateral mechanical allodynia throughout the timecourse tested, in agreement with prior studies [22-24]. Again, neither vector altered response thresholds of sham-operated rats. AAV2 appears to predominantly infect meningeal cells surrounding the CSF space, as indicated by beta-galactosidase staining of spinal tissues from rats injected with AAV2-LacZ. This pattern of meningeal staining is in accordance with prior studies of intrathecal adenovirus administration [25].

IL-10 is only one of many endogenous anti-inflammatory cytokines. In addition to IL-10, the anti-inflammatory cytokine family also includes IL-4, IL-11, and IL-13 [28,29]. Leukemia inhibitory factor, interferon-alpha, IL-6, and transforming growth factor-beta are categorized as either anti-inflammatory or pro-inflammatory cytokines, under various circumstances [29-32]. Anti-inflammatory effects are also exerted by a variety of endogenous agents as well, such as IL-1 receptor antagonist, soluble and membrane-bound IL-1 decoy receptors, and soluble TNF decoy receptors [29]. Thus a number of anti-inflammatory substances exist which may potentially exhibit therapeutic effects for enhanced pain states.

IL-10 was chosen for the present study for several reasons. First, it is considered to be the most powerful anti-inflammatory cytokine, potently downregulating TNF, IL-1 and IL-6 production and release [19,29,33]. In addition, IL-10 can upregulate endogenous anti-cytokines and downregulate pro-inflammatory cytokine receptors [19,29,33]. Thus, it can counter-regulate production and function of pro-inflammatory cytokines at multiple levels. Second, simultaneous suppression of multiple pro-inflammatory cytokines, rather than targeting a single cytokine, has advantages for 2 reasons: (a) pro-inflammatory cytokines are redundant, such that blockade of a single pro-inflammatory cytokine results in its functions being taken over by other pro-inflammatory cytokines [34], and (b) TNF, IL-1 and IL-6 can vary greatly in their relative magnitude of production, dependent both upon the inciting stimulus and time. This has been observed in spinal cord under conditions of pain facilitation as well [35]. Third, acute administration of IL-10 protein has been documented in previous studies to suppress the development of spinally-mediated pain facilitation in diverse animal models, including intrathecal dynorphin, peri-sciatic snake venom phospholipase A2, and spinal cord excitotoxic injury [36-40]. As IL-10 has a very short half-life (~2 hr) in rat cerebrospinal fluid (L. He, R. Chavez and K. Johnson, unpublished observations), IL-10 gene therapy may provide an efficient means of attaining neuropathic pain control across days. Lastly, evidence to date indicates that spinal cord neurons do not express receptors for IL-10 under either basal or inflammatory conditions [41]. Therefore, in spinal cord, IL-10 may selectively target glia, without disrupting neuronal function. Indeed, the major reported effect of IL-10 on neurons is enhancement of neuronal survival, an effect thought to be indirect via the inhibition of glially-derived neuroexcitotoxic products, including pro-inflammatory cytokines [42-45].

Replication-defective adeno-associated viral vectors offer numerous advantages for use in human gene therapy. This vector rarely inserts into host DNA, thus avoiding insertional effects common to other gene therapy approaches [46]. In addition, AAV is less inflammatory than other gene delivery vectors, such as adenovirus [47]. For such reasons, adeno-associated viruses have recently attracted attention as vectors for human gene therapy [48].

However, the present report found the efficacy of AAV2 to be short-lived upon intrathecal administration. This was surprising, given the generally accepted long-term persistence of AAV transgene expression. When AAV is administered directly into spinal cord tissue or dorsal root ganglia, AAV-directed gene expression persists for at least 4–8 months [49,50]. Since AAV injection into spinal cord or dorsal root ganglia requires traumatic surgery to expose the site, such approaches are not optimal for human application. Our intent is to explore therapies with potential use in humans, and therefore an acute intrathecal injection via percutaneous lumbar puncture route of administration was used. It is possible that AAVs of different serotypes and promoters may produce far more long-lasting effects following intrathecal administration, as serotype and promoter tropism of AAV can greatly affect what cell type(s) are infected and/or express the transgene [50-53]. Indeed, our ongoing studies suggest that far longer IL-10 protein expression can be attained by altering the AAV serotype used for intrathecal gene therapy (T. Liu, R. Chavez, and K. Johnson, unpublished data). Thus, while the results with AAV2 reported in the present studies were transient, they do indicate the potential for intrathecal IL-10 gene therapy for pain.

## Conclusion

The conclusions from the present experiments are clear. First, intrathecal IL-10 gene therapy shows promise for control of neuropathic pain. This is exciting as it demonstrates that targeting products of spinal cord glial activation can produce prolonged suppression of both mechanical allodynia and thermal hyperalgesia. Second, neuropathic pain was not only prevented but also reversed by intrathecal IL-10 gene therapy. This is important, as it implies that spinal cord glial activation contributes to both the initiation and maintenance of neuropathic pain. Third, intrathecally administered AAV2 targets primarily meningeal cells. This may be advantageous, as infection of meningeal cells avoids retrograde transport of AAV to distant sites, as has been observed in brainstem neurons following intra-spinal cord injections [25]. Lastly, it is anticipated that the duration and potency of AAV-IL-10 gene therapy can be improved by careful selection of the optimal AAV serotype.

## Methods

### Subjects

Pathogen-free adult male Sprague-Dawley rats (300–425 g; Harlan Labs) were used in all experiments. Rats were housed in temperature (23+/-3°C) and light (12:12 light: dark; lights on at 0700 hr) controlled rooms with standard rodent chow and water available *ad libitum*. Behavioral testing was performed during the light cycle. The Institutional Animal Care and Use Committee of the University of Colorado at Boulder approved all procedures.

### Drugs and adeno-associated viral vectors

Zymosan (yeast cell walls; Sigma Chemical Co., St. Louis, MO) was made fresh daily by suspension in a vehicle of incomplete Freund's adjuvant (Sigma Chemical Co., St. Louis, MO). The final concentrations were 0 (vehicle control), 0.08, or 3.2 ug/ul delivered peri-sciatically in 50 ul as described previously [10].

A replication-defective adeno-associated virus (AAV) expression vector (serotype II) containing the cDNA encoding for rat IL-10 (r-IL-10) was created, packaged and purified at the Powell Gene Therapy Center, University of Florida at Gainesville, as previously described [54]. Briefly, co-transfection was conducted with the proviral cassette with plasmid (pDG) that provides the AAV rep and cap genes in trans as well as adenoviral genes E2a, E4 and VA. The E1a and E1b genes were in the complimentary cell line, HEK 293. The vector cassette containing the cDNA encoding rat IL-10 (AAV2-r-IL-10) was driven by the hybrid cytomegalovirus (CMV) enhancer/chicken beta actin promoter/hybrid intron (pTR2-CB-rIL-10). The control AAV (AAV2-GFP) was an analogous AAV expression vector in which the CMV enhancer/chicken beta actin promoter directs the expression of the reporter gene encoding jellyfish green fluorescent protein (GFP). Viral titers were determined by infectious center assay as previously described [54]. Here, viral titers were approximately 1.7 × 10^11 infectious particles/ml (total dose given was approximately 8.5 × 10^8 infectious particles in 5 ul) and 1.69 × 10^11 infectious particles/ml (total dose given was 8.45 × 10^8 infectious particles in 5 ul) for AAV2-r-IL-10 and AAV2-GFP, respectively.

A replication-defective AAV expression vector (serotype II) containing the cDNA encoding LacZ, driven by the CMV promoter that directs the expression of beta-galactosidase was obtained from Avigen (Alameda, CA. USA). Briefly, HEK 293 cells were co-transfected by the calcium phosphate method with pAAV4.6CMV-lacZ (a transgene vector encoding beta-galactosidase), an adenovirus helper plasmid and an AAV plasmid encoding the AAV2 rep and cap genes as described [55]. For each transfection, 10 ug of each plasmid was used. The cells were harvested after 48 hrs, centrifuged, and resuspended in Tris-buffered saline (TBS). Cell lysates were collected after three freeze-thaw cycles (alternating between dry ice-ethanol and 37°C baths). The lysates were made free of debris by centrifugation. This supernatant was precipitated with PEG (8000) and the AAV pellet was fractionated on a CsCl gradient overnight. The AAV band containing functional viral particles was removed and precipitated again. The final material was resuspended in a buffer containing TBS and Pluronic F-68 (0.01%). Viral titers were approximately 4.1 × 10^10 vector genomes/ml (total dose given was approximately 4.1 × 10^8 vector genomes in 10 ul)

### Behavioral Measures

#### von Frey Test

The von Frey test [56] was performed within the sciatic or saphenous innervation area of the hindpaws as previously described [20,27,57,58]. Briefly, a logarithmic series of 10 calibrated Semmes-Weinstein monofilaments (von Frey hairs; Stoelting, Wood Dale, IL) was applied randomly to the left and right hind paws to determine the stimulus intensity threshold stiffness required to elicit a paw withdrawal response. Log stiffness of the hairs is determined by log_10 _(milligrams × 10). The 10 stimuli had the following log-stiffness values (values in milligrams are given in parenthesis): 3.61 (407 mg), 3.84 (692 mg), 4.08 (1,202 mg), 4.17 (1,479 mg), 4.31 (2,041 mg), 4.56 (3,630 mg), 4.74 (5,495 mg), 4.93 (8,511 mg), 5.07 (11,749 mg), and 5.18 (15,136 mg). The range of monofilaments used in these experiments (0.407–15.136 gm) produces a logarithmically graded slope. Interpolated 50% response threshold data is expressed as stimulus intensity in log_10 _(milligrams × 10). Assessments were made prior to (baseline) and at specific times after peri-sciatic and intrathecal drug administration, as detailed below for each experiment. Behavioral testing was performed blind with respect to drug administration. The behavioral responses were used to calculate the 50% paw withdrawal threshold (absolute threshold), by fitting a Gaussian integral psychometric function using a maximum-likelihood fitting method [59,60], as described in detail previously [57,58]. This fitting method allows parametric statistical analyses, as discussed previously [57,58].

#### Hargreaves Test

Thresholds for behavioral response to heat stimuli applied to each hindpaw were assessed using the Hargreaves test [61], as previously described [58]. Briefly, baseline (BL) paw withdrawal values were calculated from an average of 3–6 consecutive withdrawal latencies of both the left and right hindpaws measured during a 1 hr period. Voltage to the heat source was adjusted to yield BL latencies ranging 8–12 sec and a cut off time of 20 sec was imposed to avoid tissue damage. This procedure was followed by intrathecal injections and a timecourse of post-drug behavioral assessments, as described below. Behavioral testing was performed blind with respect to drug administration. The order of paw testing varied randomly.

### Surgery and microinjections

#### Acute lumbar punctures

An injection catheter was temporarily inserted under brief isoflurane anesthesia (1–2% in oxygen). Here, a 25 cm PE-10 catheter (attached by a 30-gauge sterile needle to a sterile, 50 ul glass Hamilton syringe) was marked 7.7–7.8 cm from the open end and placed in a sterile, dry container until the time of injection. Under light anesthesia, the dorsal pelvic area was shaved and swabbed with 70% alcohol. An 18-gauge sterile needle with the plastic hub removed was inserted between lumbar vertebrae L5 and L6. The open end of the PE-10 catheter was inserted via the 18-gauge needle and threaded to the 7.7 cm mark allowing for intrathecal PE-10 catheter-tip placement at the level of the lumbosacral enlargement. Drugs were injected over 1 min with a 1 ul pre- and post 0.9% sterile, isotonic saline flush. The PE-10 catheter was immediately withdrawn and the 18-gauge needle was removed from the L5-L6 inter-vertebral space. This acute injection method took 2–3 min to complete, and rats showed full recovery from anesthesia within 10 min. No abnormal motor behavior was observed after any injection. Lumbar puncture injections were only performed by investigators proven to have a 100% accuracy rate with the identical procedure using Evans' blue dye for injection confirmation in >12 rats.

#### Chronic peri-sciatic catheters

Peri-sciatic catheters were constructed and implanted at mid-thigh level of the left hind leg as previously described [10,20,27]. This method allowed multi-day recovery from isoflurane anesthesia prior to unilateral microinjection of an immune activator or vehicle around the sciatic nerve. This avoids the deleterious effects of anesthetics on the function of both immune [62-64] and glial cells [65-68]. In addition, this indwelling catheter method allowed peri-sciatic immune activation to be either acute (single injection of an immune activator) or chronic (repeated injections across weeks) [10]. Both methods were used in the present experiments in awake, unrestrained rats. These acute and chronic peri-sciatic microinjections over the left sciatic nerve were performed as previously described [10,20]. Catheters were verified at sacrifice by visual inspection. Data were only analyzed from confirmed sites.

#### Chronic constriction injury (CCI)

CCI was created at mid-thigh level of the left hindleg as previously described [21]. Four sterile, absorbable surgical chromic gut sutures (cuticular 4-0, chromic gut, 27", cutting FS-2; Ethicon, Somerville, NJ) were loosely tied around the gently isolated sciatic nerve under isoflurane anesthesia (Phoenix Pharm., St. Joseph, MO). The sciatic nerves of sham-operated rats were identically exposed but not ligated. Suture placements were verified at sacrifice by visual inspection. Data were only analyzed from confirmed sites.

### Histochemistry for the expression of AAV2 driven beta-galactosidase

Histochemistry for spinal cord beta-galactosidase was conducted as previously described [25] with following changes described here. Briefly, rats were deeply anaesthetized with sodium pentobarbital and transcardially perfused with 0.9% saline (5 min) followed by chilled, fresh 2% paraformaldehyde in 0.1% PBS (5 min). Whole spinal cords 3 cm rostral and 3.5 cm caudal to the injection site were collected and post-fixed in 2% paraformaldehyde for 15 min at room temperature. As a first examination of the spread of AAV2 driven beta-galactosidase, spinal cord was sectioned into 0.5–1 cm segments, collected into 12-well plates and washed (30 min each at room temperature with gentle agitation) 3 times with LacZ wash solution (0.1 M PBS, 0.1% deoxycholic acid [Sigma-Aldrich, St. Louis, MO], 0.2% IGEPAL CA-630 [Sigma-Aldrich, St. Louis, MO] and 2 mM MgCl_2 _in distilled water). Tissues were transferred to clean 12-well plates containing LacZ stain solution (5 mM K-ferri/ferrocyanide [Sigma-Aldrich, St. Louis, MO], 10% X-gal [Sigma-Aldrich, St. Louis, MO] in LacZ wash solution) and incubated in the dark for 3 hr and 45 min at 37°C followed by overnight incubation at 4°C, post-fixed for 24 hr in 4 % paraformaldehyde at 4°C and stored in 70% ethanol at 4°C. After inspection of beta-galactosidase staining in rostro-caudally re-constructed spinal cords of AAV2 injected and control rats, spinal segments (control and LacZ treated) were sliced at 45-micron sections and photomicrographed according to methods previously described [57]. Briefly, large segments were cryoprotected (30% sucrose in phosphate buffer overnight), frozen embedded in OCT compound (Tek, Ted Pelli Inc, Redding, CA), cryostat-sectioned at 25 microns and thaw- mounted, (0.5% gelatin-treated glass slides; Fisherbrand Superfrost Plus Slides; Fisher Scientific, Pittsburgh, PA). Spinal cord segments from the injection site (L3–L6) to 1.5 cm rostral to the injection site, and at the most caudal area of spinal cord (C2–C4) were collected because these areas best represent the degree of spread of beta-galactosidase expression from the injection site. The thaw mounted, cryostat sections (3–4 per slide) were viewed for beta-galactosidase expression with an Olympus bright-field microscope (model BX61). Images were collected with an Olympus Magnafire camera coupled to a Dell computer equipped with Olympus MagnaFire SP for windows software.

### Data Analysis

All statistical comparisons were computed using Statview 5.0.1 for the Macintosh. Data from the von Frey test were analyzed as the interpolated 50% threshold (absolute threshold) in log base 10 of stimulus intensity (monofilament stiffness in milligrams × 10). Baseline measures for both the von Frey and Hargreaves tests, and dose response effects of adenovirus, were analyzed by one-way ANOVA. Timecourse measures for each behavioral test were analyzed by repeated measures ANOVAs followed by Fisher's protected least significant difference posthoc comparisons, where appropriate.

## List of Abbreviations

AAV2 – adeno-associated virus, serotype II

AAV2-r-IL-10 – adeno-associated virus (serotype II) encoding for rat interleukin-10

ANOVA – analysis of variance

BL – baseline

CCI – chronic constriction injury

CMV – cytomegalovirus

CsCl – cesium chloride

CSF – cerebrospinal fluid

GFP – green fluorescent protein

IL – interleukin

PE – polyethylene

SIN – sciatic inflammatory neuropathy

TBS – Tris-buffered saline

TNF – tumor necrosis factor

## Competing interests

Avigen is a designated collaborator on NIH grant DA018156 (Cutting Edge Biomedical Research Award, Phase 2) awarded to ED Milligan (Principal Investigator). Avigen has provided additional research support for this project.

Avigen authors hold stocks or shares in Avigen.

University of Colorado and University of Florida authors do not hold stocks or shares in Avigen.

The University of Colorado has applied for a patent relating to the content of the manuscript, for which the Watkins laboratory has received licensing fees.

No other financial competing interests exist.

## Authors' contributions

EDM performed surgeries, designed and performed the behavioral and anatomical studies, oversaw data entry, performed statistical analyses, and contributed to manuscript and graphics preparation; EMS performed surgeries and behavioral studies as well as participated in data entry, SJL grew and purified vectors, MC performed surgeries and behavioral studies as well as participated in data entry, LS: performed surgeries and behavioral studies as well as participated in data entry, JWF performed surgeries and tissue collections; SH performed surgeries and animal perfusions; PEC designed, created and tested the AAV2-rat IL-10 vector in a cell expression system, JRF contributed to data interpretation and manuscript preparation; TRF oversaw the design, creation and testing of the AAV2-rat IL-10 vector and provided comments on manuscript drafts, SFM assisted in manuscript preparation and consulted on experimental design, statistics, and interpretation, LAL oversaw the production of AAV and assisted in manuscript preparation and experiment interpretation, RC participated in data interpretation and manuscript preparation as well as advising on AAV2-LacZ anatomy methodology and producing the AAV2-LacZ vector, LRW consulted on experimental design and interpretation and was responsible for manuscript and figure preparation.

## Note added in proof

A previously undetected point mutation, resulting in an amino acid change (F129S), has been identified in the rat IL-10 gene expressed in this study. This mutation lies outside of identified receptor binding regions. Its effect on IL-10 bioactivity in vitro, if any, is currently under investigation  
